# Ethyl 3-methyl-4-oxo-4,5-dihydro-1*H*-pyrrolo[2,3-*d*]pyridazine-2-carboxyl­ate

**DOI:** 10.1107/S1600536809055081

**Published:** 2010-01-09

**Authors:** Shi-Quan Chen, Kai Jiang, Shi-Fan Wang

**Affiliations:** a Key Laboratory of Tropical Biological Resources of the Ministry of Education, Hainan University, Haikou 570228, People’s Republic of China; b Experimental Teaching Center of Marine Biology, Hainan University, Haikou 570228, People’s Republic of China; c School of Ocean, Hainan University, Haikou 570228, People’s Republic of China

## Abstract

The title compound, C_10_H_11_N_3_O_3_, was synthesized by the reaction of 3,5-bis­(ethoxy­carbon­yl)-2-formyl-4-methyl-1*H*-pyrrole and hydrazine hydrate. The angle between the pyrrole ring and the pyridazinone ring is 0.93 (9)°. In the crystal, inter­molecular N—H⋯O and N—H⋯N hydrogen-bond inter­actions link the mol­ecules into a two-dimensional network.

## Related literature

For the biological activity of pyrrolopyridazine compounds, see: Chen *et al.* (2006 ▶); Hu *et al.* (2004 ▶); Swamy *et al.* (2005 ▶). For bond-length data, see Allen *et al.* (1987 ▶).
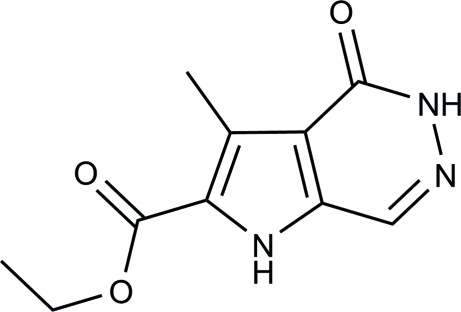


## Experimental

### 

#### Crystal data

C_10_H_11_N_3_O_3_
*M* *_r_* = 221.22
Monoclinic, 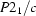

*a* = 8.0030 (16) Å
*b* = 9.774 (2) Å
*c* = 13.370 (3) Å
β = 90.17 (3)°
*V* = 1045.8 (4) Å^3^
*Z* = 4
Mo *K*α radiation
μ = 0.11 mm^−1^
*T* = 295 K
0.40 × 0.26 × 0.06 mm

#### Data collection

Bruker SMART CCD area-detector diffractometer
Absorption correction: multi-scan (*SADABS*; Sheldrick, 1996 ▶) *T*_min_ = 0.959, *T*_max_ = 0.994
6834 measured reflections
2045 independent reflections
1676 reflections with *I* > 2σ(*I*)
*R*_int_ = 0.031

#### Refinement

*R*[*F* ^2^ > 2σ(*F* ^2^)] = 0.044
*wR*(*F* ^2^) = 0.120
*S* = 1.06
2045 reflections
156 parameters
H atoms treated by a mixture of independent and constrained refinement
Δρ_max_ = 0.27 e Å^−3^
Δρ_min_ = −0.21 e Å^−3^

### 

Data collection: *SMART* (Bruker 2002 ▶); cell refinement: *SAINT* (Bruker 2002 ▶); data reduction: *SAINT*; program(s) used to solve structure: *SHELXS97* (Sheldrick, 2008 ▶); program(s) used to refine structure: *SHELXL97* (Sheldrick, 2008 ▶); molecular graphics: *PLATON* (Spek, 2009 ▶); software used to prepare material for publication: *SHELXTL* (Sheldrick, 2008 ▶).

## Supplementary Material

Crystal structure: contains datablocks global, I. DOI: 10.1107/S1600536809055081/sj2715sup1.cif

Structure factors: contains datablocks I. DOI: 10.1107/S1600536809055081/sj2715Isup2.hkl

Additional supplementary materials:  crystallographic information; 3D view; checkCIF report

## Figures and Tables

**Table 1:** Hydrogen-bond geometry (Å, °)

*D*—H⋯*A*	*D*—H	H⋯*A*	*D*⋯*A*	*D*—H⋯*A*
N3—H3⋯O1^i^	0.90 (3)	1.90 (3)	2.804 (2)	175 (2)
N1—H1⋯N2^ii^	0.86 (2)	2.08 (2)	2.925 (2)	166.2 (17)

Symmetry codes: (i) 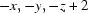; (ii) 
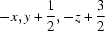
.

**Fig. 1.**
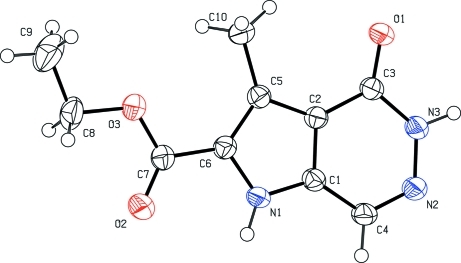
The structure of the title compound (1), showing 50% probability displacement ellipsoids and the atom-numbering scheme.

**Fig. 2.**
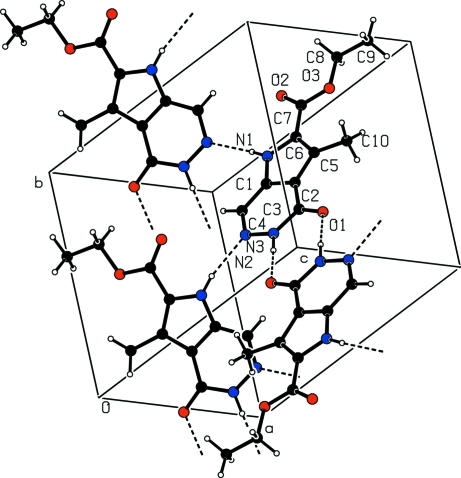
A packing diagram of compound (1) showing the chain of molecules linked by N3–H3···O1 and N1–H1···N2 hydrogen bonds. Hydrogen bonds are shown as dashed lines.

## supplementary crystallographic information

### Comment

Pyridazine and its derivatives play an important role play an important role in medicine and as pesticides. One of the main techniques to synthesize pyridazines is to react 1,4-dicarbonyl compounds with hydrazine hydrate. Recently, the synthesis of pyrrolopyridazine compounds has aroused great interest because of their significant biological activity (Chen *et al.*, 2006; Hu *et al.*, 2004; Swamy *et al.*, 2005). As part of our work to develop new types of pyrrolopyridazine compounds with potential biological activity, we report here the synthesis and structure of the title compound (1). In the molecule of compound (1), the torsion angles are N1—C1—C2—C3 179.21 (14) and N1—C1—C2—C5 0.20 (18)°. The dihedral angle between the pyrrole and pyridazinone rings is 0.93 (9)°, an indication that the pyrrolopyridazine system is reasonably planar. The C4═N2 and C3═O1 bond lengths in the molecule are 1.291 (2) and 1.2397 (19)°, respectively, showing their double-bond character (Allen *et al.*, 1987). In the crystal structure, N—H···O and N—H···N hydrogen bonds form a two-dimensional network structure, Fig. 2.

### Experimental

2.39 g (10 mmol) of the 3,5-bis(ethoxycarbonyl)-2,4- dimethyl-1(*H*)-pyrrole was added to a mixed solvent of 60 ml THF and 60 ml glacial acetic acid at room temperature under stirring until all of the solid was dissolved. Then, 60 ml water and 21.93 g (40 mmol) cerous ammonium nitrate (CAN) were added consecutively and the mixture stirred at room temperature for 1.5 h until the reaction was complete. The reaction mixture was poured into ice water and the white solid was separated (2.13 g, 84%). Recrystallization of the white solid from ethanol gave the compound 3,5-bis(ethoxycarbonyl)-2-formyl-4-methyl-1(*H*)-pyrrole.

An aqueous solution of hydrazine hydrate (80%, 0.5 ml) was added into a solution of 3,5-bis(ethoxycarbonyl)-2-formyl-4-methyl- 1(*H*)-pyrrole (0.25 g, 1.0 mmol) in glacial acetic acid (20 ml) under stirring at room temperature. The reaction mixture was refluxed for 3 h till the reaction was complete. The reaction mixture was evaporated to remove the solvent of water and acetic acid at reduced pressure to yield the title compound (1) as a white solid (0.18 g, 82%). Recrystallization of the white solid from hot ethanol yielded colorless plate-like crystals suitable for X-ray diffraction analysis.

### Refinement

The H atoms bound to N1 and N3 were located in a difference Fourier map and refined freely with isotropic displacement parameters. All other H atoms were visible in difference maps and were subsequently treated as riding atoms with distances C—H = 0.93 - 0.97 Å.  *U*_iso_(H) was set equal to *xU*_eq_(parent atom), where *x* = 1.2 - 1.5.

### Crystal data

**Table:**

C_10_H_11_N_3_O_3_	*F*(000) = 464
*M**_r_* = 221.22	*D*_x_ = 1.405 Mg m^−^^3^
Monoclinic, *P*2_1_/*c*	Mo *K*α radiation, λ = 0.71073 Å
Hall symbol: -P 2ybc	Cell parameters from 7501 reflections
*a* = 8.0030 (16) Å	θ = 2.6–26.4°
*b* = 9.774 (2) Å	µ = 0.11 mm^−^^1^
*c* = 13.370 (3) Å	*T* = 295 K
β = 90.17 (3)°	Plate, colourless
*V* = 1045.8 (4) Å^3^	0.40 × 0.26 × 0.06 mm
*Z* = 4	

### Data collection

**Table:**

Bruker SMART CCD area-detector diffractometer	2045 independent reflections
Radiation source: fine-focus sealed tube	1676 reflections with *I* > 2σ(*I*)
graphite	*R*_int_ = 0.031
φ and ω scans	θ_max_ = 26.0°, θ_min_ = 2.6°
Absorption correction: multi-scan (*SADABS*; Sheldrick, 1996)	*h* = −9→9
*T*_min_ = 0.959, *T*_max_ = 0.994	*k* = −11→12
6834 measured reflections	*l* = −16→15

### Refinement

**Table:**

Refinement on *F*^2^	Secondary atom site location: difference Fourier map
Least-squares matrix: full	Hydrogen site location: inferred from neighbouring sites
*R*[*F*^2^ > 2σ(*F*^2^)] = 0.044	H atoms treated by a mixture of independent and constrained refinement
*wR*(*F*^2^) = 0.120	*w* = 1/[σ^2^(*F*_o_^2^) + (0.0631*P*)^2^ + 0.2196*P*] where *P* = (*F*_o_^2^ + 2*F*_c_^2^)/3
*S* = 1.06	(Δ/σ)_max_ < 0.001
2045 reflections	Δρ_max_ = 0.27 e Å^−^^3^
156 parameters	Δρ_min_ = −0.21 e Å^−^^3^
0 restraints	Extinction correction: *SHELXL97* (Sheldrick, 2008), Fc^*^=kFc[1+0.001xFc^2^λ^3^/sin(2θ)]^-1/4^
Primary atom site location: structure-invariant direct methods	Extinction coefficient: 0.007 (2)

### Special details

**Table:**

Geometry. All e.s.d.'s (except the e.s.d. in the dihedral angle between two l.s. planes) are estimated using the full covariance matrix. The cell e.s.d.'s are taken into account individually in the estimation of e.s.d.'s in distances, angles and torsion angles; correlations between e.s.d.'s in cell parameters are only used when they are defined by crystal symmetry. An approximate (isotropic) treatment of cell e.s.d.'s is used for estimating e.s.d.'s involving l.s. planes.
Refinement. Refinement of *F*^2^ against ALL reflections. The weighted *R*-factor *wR* and goodness of fit *S* are based on *F*^2^, conventional *R*-factors *R* are based on *F*, with *F* set to zero for negative *F*^2^. The threshold expression of *F*^2^ > σ(*F*^2^) is used only for calculating *R*-factors(gt) *etc*. and is not relevant to the choice of reflections for refinement. *R*-factors based on *F*^2^ are statistically about twice as large as those based on *F*, and *R*- factors based on ALL data will be even larger.

### Fractional atomic coordinates and isotropic or equivalent isotropic displacement parameters (Å^2^)

**Table:**

	*x*	*y*	*z*	*U*_iso_*/*U*_eq_	
C1	0.0960 (2)	0.38216 (17)	0.86245 (11)	0.0291 (4)	
C2	0.14214 (19)	0.33040 (17)	0.95543 (11)	0.0277 (4)	
C3	0.0970 (2)	0.19196 (17)	0.97885 (11)	0.0295 (4)	
C4	0.0078 (2)	0.30214 (18)	0.79200 (12)	0.0348 (4)	
H2	−0.0209	0.3395	0.7303	0.042*	
C5	0.22621 (19)	0.43535 (17)	1.00880 (11)	0.0287 (4)	
C6	0.2266 (2)	0.54646 (17)	0.94508 (11)	0.0293 (4)	
C7	0.2897 (2)	0.68717 (18)	0.95437 (12)	0.0324 (4)	
C8	0.4347 (2)	0.8457 (2)	1.05488 (15)	0.0473 (5)	
H8A	0.3500	0.9150	1.0436	0.057*	
H8B	0.5247	0.8602	1.0076	0.057*	
C9	0.4995 (3)	0.8551 (3)	1.15900 (17)	0.0665 (7)	
H9A	0.5831	0.7860	1.1694	0.100*	
H9B	0.4094	0.8414	1.2052	0.100*	
H9C	0.5478	0.9438	1.1696	0.100*	
C10	0.2976 (2)	0.4242 (2)	1.11193 (12)	0.0380 (4)	
H10A	0.2233	0.4670	1.1587	0.057*	
H10B	0.4043	0.4690	1.1142	0.057*	
H10C	0.3111	0.3295	1.1291	0.057*	
N1	0.14838 (18)	0.51266 (15)	0.85690 (10)	0.0318 (4)	
N2	−0.03348 (19)	0.17759 (15)	0.81245 (10)	0.0362 (4)	
N3	0.01190 (19)	0.12734 (16)	0.90363 (10)	0.0349 (4)	
O1	0.12783 (16)	0.13034 (12)	1.05782 (8)	0.0397 (4)	
O2	0.27522 (18)	0.77051 (14)	0.88893 (10)	0.0506 (4)	
O3	0.36361 (16)	0.71015 (13)	1.04147 (9)	0.0409 (4)	
H1	0.132 (2)	0.565 (2)	0.8057 (15)	0.038 (5)*	
H3	−0.028 (3)	0.042 (3)	0.9145 (16)	0.067 (7)*	

### Atomic displacement parameters (Å^2^)

**Table:**

	*U*^11^	*U*^22^	*U*^33^	*U*^12^	*U*^13^	*U*^23^
C1	0.0348 (9)	0.0251 (9)	0.0274 (8)	0.0005 (7)	−0.0006 (6)	0.0008 (7)
C2	0.0313 (8)	0.0261 (9)	0.0256 (8)	0.0013 (7)	−0.0007 (6)	0.0005 (7)
C3	0.0342 (9)	0.0275 (9)	0.0268 (8)	−0.0004 (7)	−0.0005 (6)	0.0017 (7)
C4	0.0503 (10)	0.0287 (10)	0.0253 (8)	−0.0019 (8)	−0.0054 (7)	0.0011 (7)
C5	0.0299 (8)	0.0271 (9)	0.0290 (8)	0.0013 (6)	−0.0012 (6)	−0.0003 (7)
C6	0.0321 (8)	0.0273 (9)	0.0286 (8)	−0.0002 (7)	−0.0028 (6)	−0.0013 (7)
C7	0.0347 (9)	0.0285 (10)	0.0340 (9)	−0.0017 (7)	−0.0022 (7)	−0.0007 (7)
C8	0.0492 (11)	0.0365 (12)	0.0562 (12)	−0.0114 (9)	−0.0071 (9)	−0.0098 (9)
C9	0.0655 (14)	0.0758 (18)	0.0581 (14)	−0.0170 (13)	−0.0081 (11)	−0.0236 (13)
C10	0.0447 (10)	0.0367 (11)	0.0325 (9)	−0.0025 (8)	−0.0098 (7)	0.0021 (8)
N1	0.0432 (8)	0.0254 (8)	0.0268 (7)	−0.0024 (6)	−0.0058 (6)	0.0050 (6)
N2	0.0515 (9)	0.0298 (9)	0.0272 (7)	−0.0048 (7)	−0.0059 (6)	−0.0008 (6)
N3	0.0502 (9)	0.0255 (9)	0.0290 (7)	−0.0063 (6)	−0.0053 (6)	0.0023 (6)
O1	0.0569 (8)	0.0305 (7)	0.0317 (7)	−0.0077 (6)	−0.0101 (5)	0.0078 (5)
O2	0.0723 (10)	0.0319 (8)	0.0475 (8)	−0.0113 (6)	−0.0173 (7)	0.0092 (6)
O3	0.0514 (8)	0.0324 (8)	0.0389 (7)	−0.0092 (6)	−0.0101 (6)	−0.0006 (5)

### Geometric parameters (Å, °)

**Table:**

C1—N1	1.345 (2)	C7—O3	1.324 (2)
C1—C2	1.391 (2)	C8—O3	1.453 (2)
C1—C4	1.412 (2)	C8—C9	1.487 (3)
C2—C5	1.418 (2)	C8—H8A	0.9700
C2—C3	1.435 (2)	C8—H8B	0.9700
C3—O1	1.2396 (19)	C9—H9A	0.9600
C3—N3	1.368 (2)	C9—H9B	0.9600
C4—N2	1.291 (2)	C9—H9C	0.9600
C4—H2	0.9300	C10—H10A	0.9600
C5—C6	1.380 (2)	C10—H10B	0.9600
C5—C10	1.495 (2)	C10—H10C	0.9600
C6—N1	1.374 (2)	N1—H1	0.86 (2)
C6—C7	1.470 (2)	N2—N3	1.3626 (19)
C7—O2	1.201 (2)	N3—H3	0.90 (3)
			
N1—C1—C2	108.20 (14)	O3—C8—H8B	110.1
N1—C1—C4	130.09 (15)	C9—C8—H8B	110.1
C2—C1—C4	121.71 (16)	H8A—C8—H8B	108.4
C1—C2—C5	108.11 (15)	C8—C9—H9A	109.5
C1—C2—C3	118.14 (14)	C8—C9—H9B	109.5
C5—C2—C3	133.74 (14)	H9A—C9—H9B	109.5
O1—C3—N3	119.96 (16)	C8—C9—H9C	109.5
O1—C3—C2	126.47 (15)	H9A—C9—H9C	109.5
N3—C3—C2	113.57 (14)	H9B—C9—H9C	109.5
N2—C4—C1	120.59 (15)	C5—C10—H10A	109.5
N2—C4—H2	119.7	C5—C10—H10B	109.5
C1—C4—H2	119.7	H10A—C10—H10B	109.5
C6—C5—C2	105.10 (14)	C5—C10—H10C	109.5
C6—C5—C10	128.68 (15)	H10A—C10—H10C	109.5
C2—C5—C10	126.22 (15)	H10B—C10—H10C	109.5
N1—C6—C5	109.79 (15)	C1—N1—C6	108.79 (14)
N1—C6—C7	116.94 (14)	C1—N1—H1	123.8 (13)
C5—C6—C7	133.26 (15)	C6—N1—H1	127.4 (13)
O2—C7—O3	124.61 (16)	C4—N2—N3	117.50 (14)
O2—C7—C6	122.72 (16)	N2—N3—C3	128.49 (16)
O3—C7—C6	112.68 (14)	N2—N3—H3	112.6 (14)
O3—C8—C9	107.89 (17)	C3—N3—H3	118.8 (14)
O3—C8—H8A	110.1	C7—O3—C8	115.92 (14)
C9—C8—H8A	110.1		
			
N1—C1—C2—C5	0.20 (18)	C10—C5—C6—C7	−1.4 (3)
C4—C1—C2—C5	−179.24 (15)	N1—C6—C7—O2	−0.1 (3)
N1—C1—C2—C3	179.22 (14)	C5—C6—C7—O2	−178.97 (18)
C4—C1—C2—C3	−0.2 (2)	N1—C6—C7—O3	−179.76 (14)
C1—C2—C3—O1	−179.84 (16)	C5—C6—C7—O3	1.3 (3)
C5—C2—C3—O1	−1.1 (3)	C2—C1—N1—C6	−0.42 (18)
C1—C2—C3—N3	0.1 (2)	C4—C1—N1—C6	178.97 (17)
C5—C2—C3—N3	178.82 (16)	C5—C6—N1—C1	0.49 (19)
N1—C1—C4—N2	−179.01 (17)	C7—C6—N1—C1	−178.65 (14)
C2—C1—C4—N2	0.3 (3)	C1—C4—N2—N3	−0.3 (2)
C1—C2—C5—C6	0.09 (18)	C4—N2—N3—C3	0.2 (3)
C3—C2—C5—C6	−178.72 (17)	O1—C3—N3—N2	179.87 (16)
C1—C2—C5—C10	−179.86 (15)	C2—C3—N3—N2	−0.1 (2)
C3—C2—C5—C10	1.3 (3)	O2—C7—O3—C8	−1.8 (3)
C2—C5—C6—N1	−0.35 (18)	C6—C7—O3—C8	177.84 (15)
C10—C5—C6—N1	179.60 (15)	C9—C8—O3—C7	175.68 (16)
C2—C5—C6—C7	178.60 (17)		

### Hydrogen-bond geometry (Å, °)

**Table:**

*D*—H···*A*	*D*—H	H···*A*	*D*···*A*	*D*—H···*A*
N3—H3···O1^i^	0.90 (3)	1.90 (3)	2.804 (2)	175 (2)
N1—H1···N2^ii^	0.86 (2)	2.08 (2)	2.925 (2)	166.2 (17)

Symmetry codes: (i) −*x*, −*y*, −*z*+2; (ii) −*x*, *y*+1/2, −*z*+3/2.
